# Paternal age and telomere length in twins: the germ stem cell selection paradigm

**DOI:** 10.1111/acel.12334

**Published:** 2015-04-10

**Authors:** Jacob B Hjelmborg, Christine Dalgård, Massimo Mangino, Tim D Spector, Ulrich Halekoh, Sören Möller, Masayuki Kimura, Kent Horvath, Jeremy D Kark, Kaare Christensen, Kirsten O Kyvik, Abraham Aviv

**Affiliations:** 1 Department of Epidemiology, Biostatistics and Biodemography, Institute of Public Health, University of Southern DenmarkOdense, 5000, Denmark; 2 The Danish Twin Registry, University of Southern DenmarkOdense, 5000, Denmark; 3 Department of Environmental Medicine, Institute of Public Health, University of Southern DenmarkOdense, 5000, Denmark; 4 Department of Twin Research and Genetic Epidemiology, King’s College LondonLondon, UK; 5 Center of Human Development and Aging, Rutgers, The State University of New Jersey, New Jersey Medical SchoolNewark, NJ, 07103, USA; 6 Epidemiology Unit, Hebrew University-Hadassah School of Public Health and Community MedicineJerusalem, 91120, Israel; 7 Institute of Regional Health Services Research, University of Southern Denmark and Odense Patient data Explorative Network (OPEN), Odense University HospitalOdense, Denmark

**Keywords:** telomeres, twins, father’s age, germ line, heritability, leukocytes

## Abstract

Telomere length, a highly heritable trait, is longer in offspring of older fathers. This perplexing feature has been attributed to the longer telomeres in sperm of older men and it might be an ‘epigenetic’ mechanism through which paternal age plays a role in telomere length regulation in humans. Based on two independent (discovery and replication) twin studies, comprising 889 twin pairs, we show an increase in the resemblance of leukocyte telomere length between dizygotic twins of older fathers, which is not seen in monozygotic twins. This phenomenon might result from a paternal age-dependent germ stem cell selection process, whereby the selected stem cells have longer telomeres, are more homogenous with respect to telomere length, and share resistance to aging.

As expressed in leukocytes, telomere length (TL) is highly heritable (Slagboom *et al*., 1994; Broer *et al*., 2013). Leukocyte TL (LTL) in the offspring positively correlates with the father’s age at the time of the offspring’s birth (De Meyer *et al*., 2007; Kimura *et al*., 2008; Broer *et al*., 2013). Thus, older paternal age at conception (PAC) of the offspring is associated with a longer offspring’s LTL. The mechanisms of the PAC effect are poorly understood. Paradoxically, while LTL (Aubert *et al*., 2012) and TL in other somatic cells (Daniali *et al*., 2013) undergo progressive age-dependent shortening, TL is longer in sperm of older men (Allsopp *et al*., 1992; Baird *et al*., 2006; Kimura *et al*., 2008; Aston *et al*., 2012).

The longer TL in sperm of older men might be attributed to telomerase. During extra-uterine life, while the activity of telomerase is repressed in somatic tissues, it is robust in the testes, presumably due to its activity in the male germ stem cells (GSCs) (Wright *et al*., 1996). Accordingly, the longer LTL in offspring of older fathers might reflect an age-related telomere elongation in the father’s sperm. The sperm telomere elongation is apparently transmitted to the offspring in allele-specific mode, according to Mendelian principles (Baird *et al*., 2003; Graakjaer *et al*., 2006). However, the exact mechanisms whereby older fathers endow their offspring with longer telomeres are not clear.

Based on two independent twin studies (see Supporting Information for Experimental Procedures and subject characteristics), we offer a potential mechanism whereby PAC might influence the offspring’s TL, measured by Southern blots (Kimura et al, 2010). PAC and maternal age at conception (MAC) were highly correlated both for the discovery sample, that is, Danish twins (*r* = 0.83, *P* < 0.0001), and for the replication sample, that is, UK twins (*r* = 0.72, *P* < 0.0001), who were all women. For this reason, the association of LTL in the twins with MAC was also modeled.

Multiple regressions of the joint effects of sex, age, and PAC on the offspring’s LTL showed that LTL was positively associated with PAC (Danish twins, beta = 0.015, *P* < 0.001; UK twins, beta 0.012, *P* < 0.001), MAC (Danish twins, beta = 0.017, *P* < 0.001; UK twins, beta 0.011, *P* < 0.01) and decreased with age (Danish twins, beta = −0.023, *P* < 0.001; UK twins, beta = −0.022, *P* < 0.001). In addition, women showed a longer LTL than men (Danish twins, beta = −0.157, *P* = 0.01). The variance in offspring LTL did not change significantly with older PAC for the Danish and UK cohorts. Notably, due to the high correlation between PAC and MAC, the PAC effect on the offspring’s LTL was difficult to distinguish statistically from that of MAC. We infer, nonetheless, that because TL is longer in sperm of older men (Kimura *et al*., 2008; Aston *et al*., 2012), the PAC/MAC effect on the offspring’s LTL stems largely from the father’s germ line rather than from that of the mother.

Within-pair correlations in LTL by PAC for the Danish twins and UK twins are shown in Fig.1. There were too few twin pairs with PAC ≥ 40 years (12 MZ and 16 DZ for the Danish twins; 13 MZ and 35 DZ for the UK twins). Therefore, estimates displayed in Fig.1 of the within-pair correlation in LTL by PAC were restricted to PAC < 40 years. In the MZ twins, these correlations were largely stable at 80–90% for both the Danish and UK twins. However in the DZ twins, for PAC, these correlations increased from 40–50% to 65–75% for both the Danish twins and the UK twins, indicating less variation in LTL within DZ pairs with increasing PAC.

**Fig 1:**
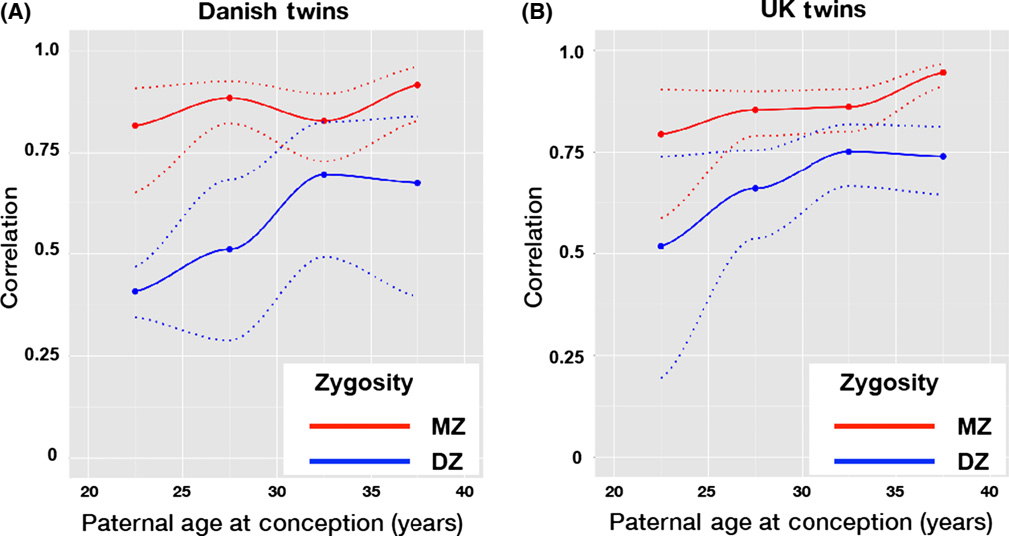
Within-pair correlation in Leukocyte telomere length (LTL) by paternal age at conception (PAC). (A) monozygotic (MZ) and dizygotic (DZ) Danish twins; (B) MZ and DZ UK twins. Dotted lines denote 95% confidence intervals.

The question then is whether the increased resemblance in LTL between DZ twins of older fathers relates in some way to the phenomenon of TL elongation with age in sperm of older men and the longer LTL in offspring of older fathers? Previous studies offered two potential though not mutually exclusive explanations for the longer TL in sperm of older men (Kimura *et al*., 2008; Aston *et al*., 2012). The first explanation is that telomerase activity slightly ‘overshoots’ with each replicative cycle of the male GSCs. The numerous replications of the male GSCs lead, therefore, to progressive lengthening of sperm TL. The second potential explanation is that a process of selection takes place with advancing age in the male GSCs, such that those GSCs that survive the effect of aging are more likely to have a longer TL. Thus, in older men, sperm with a longer TL might arise from a resilient subset of surviving GSCs, which also have robust telomerase activity.

Support of this latter concept comes from studies in model organisms and in humans. For instance, in *Drosophila melanogaster*, the testes of males display an aging-related loss of GSCs from niches, which are then repopulated through what appears to be clonal expansion of the surviving GSCs (Wallenfang *et al*., 2006). Such surviving GSCs might be more aging-resilient than those that have vanished. In humans, there is evidence of positive selection of GSCs with replicative advantage due to disease-causing mutations (Momand *et al*., 2013).

That being said, an increased mutation load with age in the male germ line is unlikely to explain the PAC effect on the offspring’s LTL. As age-dependent mutations in the male germ line are extremely rare (an average of 2.1 mutations throughout the human genome for each year of increased PAC) (Kong *et al*., 2013), they might drastically impact a trait in only a very small subset of the offspring. If this were the case, the offspring of older fathers would display an increase in LTL variance due to a small subset of offspring with extremely long LTLs. However, the PAC effect is expressed as a shift to a longer LTL, on average, in the entire offspring population without evidence of increased LTL variance.

Given that sperm are derived from different GSCs, the model of selection and clonal expansion of surviving GSCs predicts that: (i) both DZ and MZ twins, born to older fathers, would display a longer LTL; (ii) as MZ twins are the product of one sperm (and one egg), their LTL resemblance would be largely unaffected by the father’s age; and (iii) when born to an older father, DZ twins, who are the product of two sperm (and two eggs), are likely to display more LTL resemblance. Such a putative rise in the resemblance of LTL between DZ twins with older PAC might be due to a higher probability that the two sperm that successfully fertilized the two eggs were from a more homogeneous GSC lineage reflecting clonal expansion of surviving GSCs in older fathers (Fig.2). The GSC selection/clonal expansion model offers an additional explanation for the longer telomeres in both MZ and DZ twins of older fathers. As clonal expansion entails more GSC replication, it is anticipated that this process would result in further telomere elongation, based on the premise that telomerase ‘overshoots’ with each replication of male GSCs.

**Fig 2:**
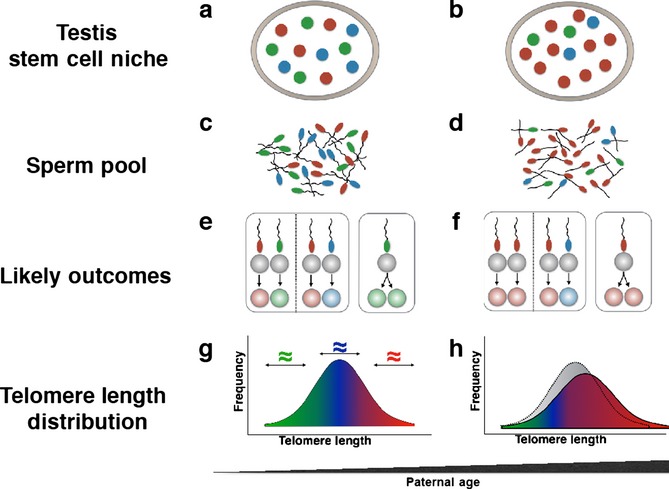
A model of selection and clonal expansion germ stem cells (GSCs) and sperm of older versus young fathers. Panels on the left apply to putative scenarios of young fathers; panels on right apply to putative scenarios of older fathers. Panels a and b show transition in the relative numbers of GSCs in older versus young fathers, based on three lineages of GSCs, where green = GSCs with short telomeres, blue = GSCs with intermediate telomeres and red = GSCs with long telomeres. For the young fathers, each of three GSC lineages is represented by four GSCs. For the older fathers, the green and blue GSC lineages are each represented by two GSCs, while the red GSC lineage is represented by eight GSCs. The assumption here is that half of the green and blue GSCs did not survive the aging process, while the red GSCs, with replicative advantage perhaps because relatively long telomeres, underwent clonal expansion. Panels c and d show an increase in the number of sperm derived from the red GSC lineage in the older father. Panels e and f show an increase in the probability that the DZ twins of the older father will be the products of two red sperm derived from the red GSC lineage. This will be expressed as an increase in telomere length in co-twins and an increase in telomere length equivalence between the DZ co-twins. As MZ twins are the product of one sperm, their telomere length is likely to increase in older fathers because of increased probability that they are the product of a red sperm, but without an apparent change in their telomere length equivalence with increasing fathers’ age. Panels g and h show the shift toward longer telomeres in GSCs of the older versus young fathers due to increased GSCs of red lineage. Notably, this model does not necessarily require that GSCs of red lineage have longer telomeres from the outset. In principle, telomere length in the red GSCs might become longer in the older father because this lineage has undergone clonal expansion, which in the testis is associated with telomere elongation due to a slight ‘overshoot’ in the activity of telomerase.

That said, one cannot exclude a TL ‘ceiling effect’ as a potential explanation for the increased LTL resemblance in DZ twins of older fathers. In principle, sperm telomeres might not lengthen beyond a finite threshold. Under such a scenario, the variance in sperm TL would decline with age. Scatter plots in previous studies of sperm TL versus the donor’s age (Allsopp *et al*., 1992; Baird *et al*., 2006; Aston *et al*., 2012) display no evidence for a decline in sperm TL variance with age, but sample sizes were relatively small to fully exclude a ‘ceiling effect’ for sperm TL dynamics..

We therefore posit that the increased resemblance in LTL in DZ co-twins of older fathers might reflect an age-dependent positive selection of GSCs with longer telomeres. Indirect support for this notion is also provided by work that shows the presence (arguably the ‘emergence’) of sperm with longer telomeres in samples donated by older men (Kimura *et al*., 2008). Moreover, this concept is in line with the model of diversification of stem cell populations based on evolutionary principles to optimize function through selection (MacArthur, 2014). Examination of the impact of PAC/MAC on LTL of MZ and DZ newborn twins might be the next step in testing our model. Should the findings observed in adult twins also be replicated in newborn twins, this would support the hypothesis that epigenetic mechanisms that impact spermatogenesis and the intra-uterine milieu might account for the PAC effect on the offspring’s LTL.

Finally, whether through GSC selection, telomerase, or both, the PAC effect on the offspring LTL, suggests that the evolutionary force on DNA sequences by the numerous replications of the male germ line in humans (Haldane, 1937; Shimmin *et al*., 1993) might be exerted through not only mutations but also TL.

## Supporting Information

**Data S1.** Experimental Procedures. **Table S1.** Characteristics of Danish and UK twin samples.

## References

[b1] Allsopp RC, Vaziri H, Patterson C, Goldstein S, Younglai EV, Futcher AB, Greider CW, Harley CB (1992). Telomere length predicts replicative capacity of human fibroblasts. Proc. Natl. Acad. Sci. U. S. A.

[b2] Aston KI, Hunt SC, Susser E, Kimura M, Factor-Litvak P, Carrell D, Aviv A (2012). Divergence of sperm and leukocyte age-dependent telomere dynamics: implications for male-driven evolution of telomere length in humans. Mol. Hum. Reprod.

[b3] Aubert G, Baerlocher GM, Vulto I, Poon SS, Lansdorp PM (2012). Collapse of telomere homeostasis in hematopoietic cells caused by heterozygous mutations in telomerase genes. PLoS Genet.

[b4] Baird DM, Rowson J, Wynford-Thomas D, Kipling D (2003). Extensive allelic variation and ultrashort telomeres in senescent human cells. Nat. Genet.

[b5] Baird DM, Britt-Compton B, Rowson J, Amso NN, Gregory L, Kipling D (2006). Telomere instability in the male germline. Hum. Mol. Genet.

[b6] Broer L, Codd V, Nyholt DR, Deelen J, Mangino M, Willemsen G, Albrecht E, Amin N, Beekman M, de Geus EJ, Henders A, Nelson CP, Steves CJ, Wright MJ, de Craen AJ, Isaacs A, Matthews M, Moayyeri A, Montgomery GW, Oostra BA, Vink JM, Spector TD, Slagboom PE, Martin NG, Samani NJ, van Duijn CM, Boomsma DI (2013). Meta-analysis of telomere length in 19,713 subjects reveals high heritability, stronger maternal inheritance and a paternal age effect. Eur. J. Hum. Genet.

[b7] Daniali L, Benetos A, Susser E, Kark JD, Labat C, Kimura M, Desai K, Granick M, Aviv A (2013). Telomeres shorten at equivalent rates in somatic tissues of adults. Nat. Commun.

[b8] De Meyer T, Rietzschel ER, De Buyzere ML, De Bacquer D, Van Criekinge W, De Backer GG, Gillebert TC, Van Oostveldt P, Bekaert S, Asklepios investigators (2007). Paternal age at birth is an important determinant of offspring telomere length. Hum. Mol. Genet.

[b9] Graakjaer J, Der-Sarkissian H, Schmitz A, Bayer J, Thomas G, Kolvraa S, Londoño-Vallejo JA (2006). Allele-specific relative telomere lengths are inherited. Hum. Genet.

[b10] Haldane JB (1937). The effect of variation on fitness. Am. Nat.

[b11] Kimura M, Cherkas LF, Kato BS, Demissie S, Hjelmborg JB, Brimacombe M, Cupples A, Hunkin JL, Gardner JP, Lu X (2008). Offspring’s leukocyte telomere length, paternal age, and telomere elongation in sperm. PLoS Genet.

[b12] Kimura M, Stone RC, Hunt SC, Skurnick J, Lu X, Cao X, Harley CB, Aviv A (2010). Measurement of telomere length by the Southern blot analysis of terminal restriction fragment lengths. Nat. Protoc.

[b13] Kong A, Frigge ML, Masson G, Besenbacher S, Sulem P, Magnusson G, Gudjonsson SA, Sigurdsson A, Jonasdottir A, Wong WS (2013). Rate of *de novo* mutations and the importance of father’s age to disease risk. Nature.

[b14] MacArthur BD (2014). Collective dynamics of stem cell populations. PNAS.

[b15] Momand JR, Xu G, Walter CA (2013). The paternal age effect: a multifaceted phenomenon. Biol. Reprod.

[b16] Shimmin LC, Chang BH, Li WH (1993). Male-driven evolution of DNA sequences. Nature.

[b17] Slagboom PE, Droog S, Boomsma DI (1994). Genetic determination of telomere size in humans: a twin study of three age groups. Am. J. Hum. Genet.

[b18] Wallenfang MR, Nayak R, DiNardo S (2006). Dynamics of the male germline stem cell population during aging of Drosophila melanogaster. Aging Cell.

[b19] Wright WE, Piatyszek MA, Rainey WE, Byrd W, Shay JW (1996). Telomerase activity in human germline and embryonic tissues and cells. Dev. Genet.

