# Supplementary material for: Paternal age and telomere length in twins: the germ stem cell selection paradigm
Source: Aging Cell. 2015 Apr 10;14(4):701–3. doi: 10.1111/acel.12334 (PMC4531084; doi:10.1111/acel.12334)
Supplement: Supplementary file 1 [file acel0014-0701-sd1.docx]

**Supporting Information**

**Experimental Procedures**

**Subjects:** The discovery sample comprised 137 DZ and 167 monozygotic MZ Danish twin pairs (both men and women) from the GEMINAKAR Study and the replicative sample comprised 217 DZ and 368 MZ British twin pairs (all women) participants in TwinsUK (Table 1).

The two studies received institutional review- board approvals and all participants provided written informed consent for participation in the parent study.

**LTL Analysis**: All LTL measurements were performed in the same laboratory by Southern blots of the terminal restriction fragments (Kimura et al, 2010). The inter-assay coefficient of variation of the TL measurements was 1.3% for the Danish twins and 1.0% for the British twins.

**Statistical analysis and modeling:** Examination of the effect of PAC and (MAC) on the LTL resemblance between the co-twins required intact twin pairs. However, both intact twin pairs and twins without their co-twins were used to examine the PAC (and MAC) effects on the offspring’s LTL. PAC and MAC were taken as the father’s and mother’s ages at the time of birth of their twins, although in reality parents were younger at the time of the twins’ conception. Analyses were independently performed for the Danish twins and then repeated for the UK twins.

Adjustment for within twin pair dependence was done by including a pair-specific random effect variable. The association was further examined by estimating the expected LTL by PAC and MAC separately using cubic spline additive mixed model regression (GAMM).

| **Supporting Information, Table 1.** Characteristics of Danish and UK twin samples | | | | | | | | |
| --- | --- | --- | --- | --- | --- | --- | --- | --- |
| **Cohort** | **N** | **N Pairs** | **Zygos** | **Sex** | **LTL (kb)** | **Age Range** | **PAC Range** | **MAC Range** |
| DK | 125 | 58 | DZ | male | 6.85 (0.60) | 20-58 | 20-52 | 19-41 |
| DK | 165 | 79 | DZ | female | 7.05 (0.70) | 20-54 | 21-58 | 18-41 |
| DK | 163 | 77 | MZ | male | 6.93 (0.65) | 20-57 | 21-48 | 18-41 |
| DK | 188 | 90 | MZ | female | 7.11 (0.65) | 19-64 | 21-50 | 18-43 |
| UK | 622 | 217 | MZ | female | 6.88 (0.63) | 18-76 | 17-57 | 18-41 |
| UK | 947 | 368 | DZ | female | 7.04 (0.67) | 18-72 | 18-62 | 16-43 |

N= number of twins, N Pairs= number of intact twin pairs, Zygos= zygosity, Age= age (in years) of the twins, PAC= paternal age (in years) at conception, MAC= maternal age (in years) at conception, DK= Denmark, UK= United Kingdom; LTL data are presented as mean (SD).
